# CAUSALITY-INFORMED REPRODUCTIVE CLOCKS REFLECT IMPACT OF CHILDHOOD MALTREATMENT ON EPIGENETIC AGING

**DOI:** 10.1093/geroni/igae098.2288

**Published:** 2024-12-31

**Authors:** Qiaofeng Ye, Idan Shalev

**Affiliations:** The Pennsylvania State University, University Park, Pennsylvania, United States; The Pennsylvania State University, University Park, Pennsylvania, United States

## Abstract

Machine-learning-based epigenetic clocks have been widely used to predict an organism’s age, pace of aging, and mortality. Here, we report the development of two novel reproductive clocks to reflect biological aging relative to menarche (MenarcheClock) or menopause (MenopauseClock) in females. First, Mendelian randomization was employed to identify CpG sites with significant causal effects on age at menarche or menopause. Next, models of MenarcheClock and MenopauseClock were trained using elastic net regression with array-measured DNA methylation of buccal cells from 540 females aged 0.2-18.94 years. In total, 186 and 141 CpG sites were selected into the MenarcheClock and the MenopauseClock, respectively, which had little overlap with published epigenetic clocks. Based on the Developmental Origin of Health and Disease model and evolutionary theories of aging, we further examined the impact of childhood maltreatment, race, ethnicity, and family income on deviations of the two clocks in 187 girls aged 8-14 years. Clock deviations were calculated by residualizing the clock estimates from chronological age, with positively greater deviations indicating expedited menarche or menopause. Preliminary analyses indicated that exposure to childhood maltreatment, including physical and sexual abuse and neglect, was associated with greater MenarcheClock deviation, controlling for BMI, race/ethnicity, income, and immune cell proportion. African American girls had greater MenopauseClock deviation than White peers. Our study demonstrated the feasibility of utilizing causality information based on Mendelian randomization to develop epigenetic clocks indicating reproductive aging. Findings also support the theory that adverse exposure during early development leads to earlier sexual maturation to increase reproductive success.

